# The diagnosis of hypertrophic cardiomyopathy by cardiovascular magnetic resonance

**DOI:** 10.1186/1532-429X-14-17

**Published:** 2012-02-20

**Authors:** Radwa A Noureldin, Songtao Liu, Marcelo S Nacif, Daniel P Judge, Marc K Halushka, Theodore P Abraham, Carolyn Ho, David A Bluemke

**Affiliations:** 1 Radiology and Imaging Sciences, National Institutes of Health Clinical Center, Bethesda, MD, USA; 2 Molecular Biomedical Imaging Laboratory, National Institute of Biomedical Imaging and Bioengineering, Bethesda, MD, USA; 3 Division of Cardiology, Department of Medicine, Johns Hopkins University, Baltimore, MD, USA; 4 Department of Pathology, Johns Hopkins University, Baltimore, MD, USA; 5 Cardiovascular Division, Brigham and Women's Hospital, Boston, MA, USA

## Abstract

Hypertrophic cardiomyopathy (HCM) is the most common genetic disease of the heart. HCM is characterized by a wide range of clinical expression, ranging from asymptomatic mutation carriers to sudden cardiac death as the first manifestation of the disease. Over 1000 mutations have been identified, classically in genes encoding sarcomeric proteins. Noninvasive imaging is central to the diagnosis of HCM and cardiovascular magnetic resonance (CMR) is increasingly used to characterize morphologic, functional and tissue abnormalities associated with HCM. The purpose of this review is to provide an overview of the clinical, pathological and imaging features relevant to understanding the diagnosis of HCM. The early and overt phenotypic expression of disease that may be identified by CMR is reviewed. Diastolic dysfunction may be an early marker of the disease, present in mutation carriers prior to the development of left ventricular hypertrophy (LVH). Late gadolinium enhancement by CMR is present in approximately 60% of HCM patients with LVH and may provide novel information regarding risk stratification in HCM. It is likely that integrating genetic advances with enhanced phenotypic characterization of HCM with novel CMR techniques will importantly improve our understanding of this complex disease.

## Introduction

Hypertrophic cardiomyopathy (HCM) is the most common heritable cardiovascular disorder, with a prevalence of 1:500 in the general population. It is also the most common cause of sudden cardiac death (SCD) in young individuals and young athletes [1,2]. HCM is an autosomal dominant disease caused by mutations in genes encoding sarcomere proteins [1]. Autosomal recessive, X-linked, and mitochondrial (matrilinear) patterns of inheritance also occur [1,3]. The penetrance of left ventricular hypertrophy (LVH) is highly age-dependent and incomplete [4]. Although the most common expression of HCM is during adolescence [5,6], patients may present at any point in life. Clinical screening of first-degree relatives is recommended annually during adolescence (age 12-18) [7]. Adult relatives of HCM affected individuals beyond age 18 may be screened at 5 year intervals.

The annual mortality rate of HCM ranges from < 1% in the general community to 3-6% in tertiary referral centers [1,8]. Current risk factor stratification for SCD considers multiple factors, including nonsustained ventricular tachycardia, syncope, exercise blood pressure response, family history of sudden death, high risk genetic mutations as considered in individual patients [7,9,10]. On the imaging level, risk factors for SCD in HCM include left ventricular (LV) wall thickness and left ventricular outflow tract (LVOT) obstruction (Table 1).

**Table 1:** Major risk factors for the assessment of sudden cardiac death in hypertrophic cardiomyopathy* (adapted from [7])

Major risk factors	Possible risk factors in individual patients
Cardiac arrest/ventricular fibrillation or spontaneously occurring and sustained VT	Atrial fibrillation
Family history of premature HCM-related sudden death	*Left ventricular outflow obstruction*
*Left ventricular thickness equal to or greater than 30 mm, especially in adolescents and young adults*	Intense physical exertion
Abnormal upright exercise blood pressure response that is attenuated or hypertensive (of greater predictive value in patients less than 50 yrs old or if hypotensive)	High risk gene mutation
Nonsustained ventricular tachycardia by Holter examination	
Unexplained syncope, especially in young patients or when recurrent or exertional	

*risk factor assessed by imaging.

The diagnosis of HCM is based largely on imaging features. Noninvasive diagnosis is based on left ventricular (LV) wall thickness ≥ 15 mm at end-diastole (frequently involving the interventricular septum) or septal to lateral wall thickness ratio higher than 1.3 in a non-dilated LV in the absence of a loading condition sufficient to cause the observed abnormality [5,11,12]. However, Maron et al., [1] have stated that virtually any LV wall thickness, even when within normal limits, can be consistent with the presence of an HCM-causing mutant gene. Further, only one major CMR manuscript has focused on establishing normal values for LV wall thickness using current steady state free precession (SSFP) pulse sequences at 1.5 T (Dawson, Dawson et al. Circ Cardiovasc Imaging 2011 [13]) in 60 men and 60 women Caucasian subjects. In that study, wall thickness was evaluated only on short axis views.

Recently there has been greatly increased use of cardiac magnetic resonance (CMR) for the diagnosis of HCM because of its precise determination of myocardial anatomy and the depiction of myocardial fibrosis. The purpose of this review is to provide the physician with a review of clinical, pathologic and genetic abnormalities relevant to understanding the potential role of CMR in HCM.

### Pathologic basis of HCM

The diagnosis of HCM was historically made by pathologists only at the untimely death of an individual [14]. Advances in cardiovascular imaging and genetics have facilitated improved diagnosis allowing for deployment of interventions such as intracardiac defibrillators that can favorably alter the natural history of the disease in high risk patients. HCM in living individuals is seen by pathologists on septal myectomy, endomyocardial biopsy, left ventricular core from assist device placement, and cardiac explant specimens. Rarely is this the primary diagnostic modality for HCM.

Grossly, the defining feature of an HCM heart is increased LV mass and thickened wall, especially the interventricular septum [15]. Grossly-evident scattered fibrosis may or may not be present. In some cases, there is a predilection for fibrosis at the junction of the septum to the anterior and posterior walls of LV. The thickened septum often bulges into LV, diminishing the LV end diastolic lumen space. When prominent this septal bulge may dynamically block left ventricular outflow. When this obstruction occurs, anterior displacement of the papillary muscles/mitral leaflets occurs and the anterior leaflet of the mitral valve rubs the septal wall upon opening resulting in endocardial thickening, noted as a pearly white alteration along the outflow tract. These abnormalities may lead to altered hydrodynamic forces resulting in systolic anterior motion (SAM) of the mitral valve and mitral leaflet-septal contact with obstructive physiology [16]. Eventually, the valve leaflets and chordae tendineae become thickened by fibrosis [14,17].

The key histologic feature of HCM is myocyte and myofibrillar disarray [15,16]. Myocyte disarray is non-linear or haphazard arrangement of the myocytes observed by light microscopy. Typically, "herringbone" or "pinwheel" configurations are noted [15]. Various papers have suggested a need for a minimal amount of myocyte disarray (usually 5 or 10%) to make the diagnosis of HCM [18,19]. This is because rare myocyte disarray can occur in other diseases or even in normal hearts where two muscle bundles come together [15].

In addition to disarray, three other non-specific findings are generally noted in HCM. One is obvious myocyte hypertrophy with nuclear enlargement, angulated nuclear borders and hyperchromasia. A second is a marked increase in both interstitial and replacement fibrosis that may be patchy of diffuse, mainly in the septal region [20]. The third is dysplasia of small arteries, seen as medial and intimal smooth muscle cell proliferation with luminal narrowing [21]. Small vessels are encased by dense perivascular collagen and also contain increased collagen within the media. Reduced arteriolar density may lead to small-vessel intramural coronary artery disease (SICAD) as an early manifestation of HCM [22]. This dysplasia is more prevalent in areas of replacement fibrosis. This suggests reduction in blood flow contributes to regional myocyte loss as a substrate for ventricular arrhythmia and subsequently SCD [1,21]. Taken together, mid-septal myocyte disarray with myocyte hypertrophy, fibrosis, dysplastic small coronary arteries, and endocardial thickening are the histopathologic hallmarks of HCM.

### Morphologic variants identified by noninvasive imaging

Multiple morphological variants of HCM have been described that can be identified by cardiac magnetic resonance (CMR). These are detailed below:

#### Asymmetric HCM with sigmoid septal contour ("septal HCM")

**Figure 1:**
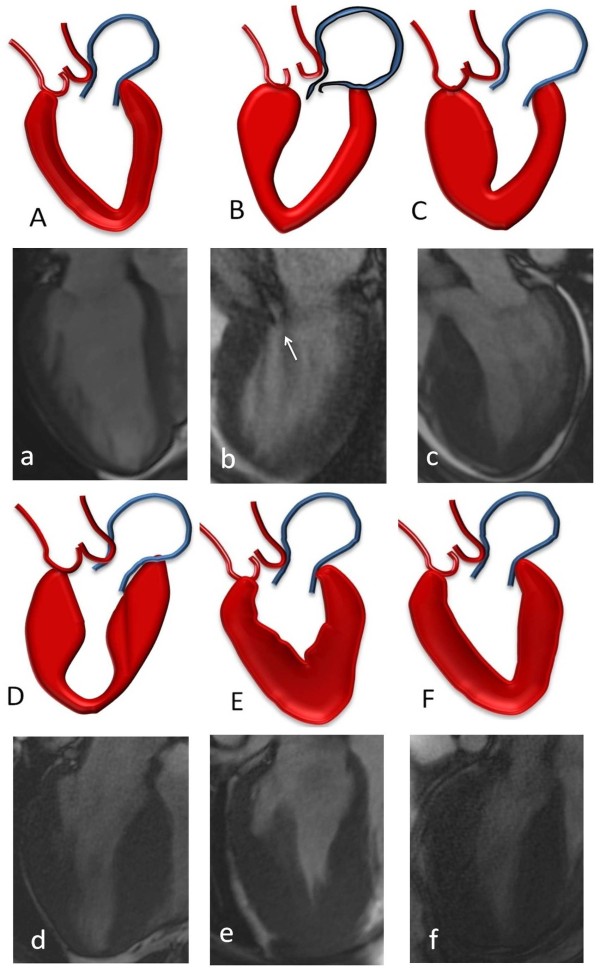
**Left ventricular patterns in HCM, each drawing is accompanied by its corresponding image, (A, a) normal LV, (B, b) sigmoid septum showing SAM of mitral valve (white arrow), (C, c) reversed septal contour, note that there is no signs of LVOT, (D, d) mid ventricular hypertrophy, (E, e) Apical HCM, (F, f) symmetric HCM**.

This is the most common morphologic presentation HCM, accounting for about two thirds of the spectrum [23,24]. Hypertrophy of the anteroseptal myocardium results in sigmoidal contour of the septum [15,25,26]. As indicated above, these abnormalities may lead to altered hydrodynamic forces resulting in SAM of the mitral valve and mitral leaflet-septal contact with obstructive physiology [16]. This results in subaortic obstruction and a concomitant mitral regurgitant jet (Figure 1 B, b) directed posteriorly into the left atrium (LA) due to incomplete leaflet apposition. Peak instantaneous outflow tract gradients can be estimated using phase-contrast CMR [25,27]. Anterior and posterior mitral valve leaflet length in HCM are greater than in control subjects by CMR (26 ± 5 versus 19 ± 5 mm, P < 0.001; and 14 ± 4 versus 10 ± 3 mm, P < 0.001, respectively). This anatomic feature has been postulated to be responsible for LVOT obstruction [28].

#### *Asymmetric HCM *with reversed septal contour

In this form, the septum hypertrophies as a reversed S-type curve (Figure 1 C, c) that does not cause LVOT obstruction [24,29,30]. LV mass may not be increased in patients with asymmetric HCM, especially with focal hypertrophy involving less than two LV segments [24,31]. Subtle evidence of regional wall hypocontractility may be present despite a normal LV ejection fraction (EF). Systolic function declines as LVH becomes more severe [24,30].

Asymmetric HCM is identified on short axis images characterized by a septal: free wall thickness ratio > 1.3 [15].

#### HCM with mid-ventricular obstruction (with or without a LV apical diverticulum)

In this variant, there is marked mid-ventricular hypertrophy (Figure 1 D, d) together with mid-cavity narrowing giving a 'dumb-bell' configuration of LV [27,29]. In severe cases of narrowing, apical dilatation may occur. The apical dilatation is thought to result from the generation of increased systolic pressures within the cardiac apex due to mid-ventricular obstruction [15,27].

In approximately 10% of patients, there is progression to a "burned out apex" with apical aneurysm formation and late gadolinium enhancement (LGE) after gadolinium administration [32]. This appearance is thought to result from ischemia resulting from reduced capillary density, hyperplasia of the arterial media, increased perivascular fibrosis and myocardial bridging [15,33].

#### Apical HCM

In apical HCM, there is obliteration of LV cavity at the apex, giving a characteristic spade-like configuration of the cavity [34] seen on vertical long axis views (Figure 1 E, e). An apical wall thickness of > 15 mm or a ratio between apical and basal LV wall thicknesses ≥ 1.3-1.5 are characteristically present [35,36]. This variant is more frequent in the Japanese population (~25% of HCM patients) compared to western countries (~2% of HCM patients) [24,35].

CMR is particularly helpful in identifying apical HCM. In 10 patients with apical HCM by CMR (including one with LV wall thickness of 28 mm), echocardiograms were consistently negative [35]. Other comparisons of CMR to echocardiography for HCM diagnosis are shown in Table 2.

**Table 2:** Accuracy of CMR for detection of HCM in comparison to 2-dimensional echocardiography

Authors	Year	Journal	Number of patients	% of HCM patients detected by MRI only	Location of CMR detected abnormality
Maron et al.[24]	2009	Journal of the American College of Cardiology	333	12%	Anterolateral free wall, posterior portion of septum, LV apex
Maron et al.[108]	2007	Circulation	2	100%	Anterior free wall
Rickers et al.[23]	2005	Circulation	48	6%	Anterolateral free wall
Moon et al. [35]	2004	Heart	10	100	Apical
Pons-Llado et al.[109]	1997	Am. Journal of Cardiology	30	Echocardiography underestimated wall thickness	Anterior basal, septal anterior mid-ventricular, lateral mid-ventricular
Posma et al. [110]	1996	American Heart Journal	52	Echocardiography underestimated wall thickness in 37%.	Anterobasal septum, anterolateral wall, posteroseptal wall, apical septum, posterior wall

#### Symmetric (Concentric) HCM

In up to 42% of HCM cases, hypertrophy symmetrically involves the ventricular wall with no regional preferences (Figure 1 F, f). LV cavity dimensions are reduced in a concentric fashion [26,29]. Symmetric LVH in the absence of hypertension or aortic stenosis may also be present in a variety of disorders, including amyloidosis, sarcoidosis and Fabry's disease. Hypertrophy in the athlete's heart is typically relatively modest, LV cavity size is normal to enlarged (as compared to the typically small LV cavity seen in HCM), and there is no evidence of diastolic dysfunction nor LGE. These features help to distinguish athlete's heart from symmetric HCM [8].

#### Focal HCM

Patients with HCM occasionally present with focal, mass-like thickening of LV. In these cases, CMR may help distinguish HCM from other cardiac masses using CMR tagging by spatial modulation of magnetization (SPAM)[37]. Tag lines show evidence of myocardial contractility in HCM, whereas neoplastic masses should not be contractile [38]. In addition, the signal intensities of tumor-related masses in the myocardium on spin echo CMR, first-pass perfusion and LGE-CMR are frequently different from normal myocardium [29]. The CMR characteristics of tumor-like HCM more closely resemble that of adjacent normal myocardium.

#### Right ventricular (RV) involvement in HCM

RV hypertrophy has been reported in approximately 18% of HCM patients [24,29]. This typically involves the mid-to-apical portion of the RV. Severe involvement of the RV may result in RV outflow obstruction or reduced RV diastolic filling [15].

### Genotype-phenotype correlations in HCM

Although several echocardiographic studies have evaluated the relationship between various morphologic subtypes and sarcomeric mutation [39,40], such relationships are controversial and need further study. Sigmoidal HCM appears to be most common in the elderly and less likely to have an underlying sarcomere mutation.

In a cohort of 382 HCM patients, 73% of patients with sarcomeric HCM had reversed curve septum while only 10% had sigmoid septum. The incidence of SCD was found to be higher in specific *MYH7 *mutations [41]. Progression to heart failure was reported to be more commonly associated with mutations in *MYH7 *and *TNNI3 *than from other HCM mutations [42,43]. One particular actin mutation (p.Glu101Lys) has been reported in at least 5 families with either apical hypertrophy or LV noncompaction [44,45]. Approximately 3-5% of HCM patients may have multiple mutations (i.e., compound or digenic heterozygosity). These patients often have a more severe phenotype and increased incidence of SCD [46-49], suggesting a gene-dosage effect might contribute to disease severity. Most cases of compound heterozygosity involve *MYBPC3 *[46]. A recent report highlights the consequences of HCM resulting from 3 distinct mutations [49]. These investigators identified 4 such cases among 488 unrelated HCM probands (0.8%). Three of the 4 had severe disease that progressed to end-stage HCM by the fourth decade.

### Increased left ventricular mass in HCM

LVH is widely regarded as a requirement for clinical diagnosis of HCM [30,50,51]. However, recent genotype-phenotype correlations have shown that virtually any wall thickness may be found in patients with a HCM gene mutation [24]. Greater CMR defined LV mass is associated with less favorable clinical outcome. This is attributed to the relationship between LVH and both the presence of LVOT obstruction and more advanced heart failure [52,53]. Olivotto et al. [30] demonstrated that LV mass index is a more sensitive indicator for risk of death than peak wall thickness.

CMR provides better diagnostic accuracy than echocardiography in definition of the ventricular size, magnitude and distribution of hypertrophy, especially in identification of the anterolateral wall of LV [23,35,54]. Therefore, assessment of LV mass using CMR plays an important role in risk stratification of the disease.

### Assessment of diastolic function

Diastolic dysfunction (DD) is an early functional marker of HCM. DD occurs secondary to abnormal dissociation of actin and myosin filaments during the active phase of relaxation in early diastolic filling [55,56]. The assessment of diastolic function with CMR using myocardial tissue tagging has been previously reviewed [57]. Passive late diastole filling is also impaired due to increased interstitial fibrosis. Myocyte disarray may affect both ventricular relaxation and stiffness [55,58]. Shirani et al., [33] suggested that the expanded and disorganized architecture of collagen matrix may contribute to DD. This results in decreased peak filling rate (PFR) and increased time to PFR [59,60]. With diastole, prolongation of isovolumic relaxation is thought to be an early and sensitive marker of DD [61].

Tissue Doppler echocardiographic studies on genotyped subjects have demonstrated that sarcomere mutation carriers have diastolic abnormalities early in life, prior to the development of LVH. Impaired relaxation can be detected as decreased early myocardial relaxation velocities [62-64]. These studies indicate that diastolic abnormalities are an early, potentially, direct manifestation of the underlying sarcomere mutation, rather than simply a secondary consequence of altered myocardial compliance characteristics due to the hypertrophy, fibrosis and disarray that accompanies development of clinically overt disease.

Different CMR methods can be used to assess diastolic dysfunction. The first approach aims to parallel measures used by other imaging modalities [55,56]. *Ventricular time-volume curves *can provide accurate assessment of global diastolic function. From these, PFR and time to PFR can be estimated [58]. PFR may be "normalized" relative to end-diastolic volume (PFR/EDV) and to stroke volume (PFR/SV). Unfortunately, PFR is only partially representative of compliance [56,59]. *Mitral inflow velocities *(early filling "E" and atrial systolic filling" A") and pulmonary vein flow (systolic "S" and diastolic "D" velocities) derived from phase contrast CMR (PC-CMR) provide highly reproducible and accurate data. Velocity data can be used to calculate pressure gradients (ΔP) using the modified Bernoulli equation (ΔP = 4V^2^, where V = velocity) to classify DD [55]. Finally, *myocardial motion velocity *may be evaluated by PC-CMR. This approach can acquire images in multiple oblique planes, allowing the operator to choose in-plane or through-plane velocity acquisitions [55] with a high spatial and temporal resolution [65].

The second CMR approach to assess diastolic function is to directly measure myocardial diastolic relaxation. *LV strain rate *and *torsion recovery rate *are assessed using myocardial tissue tagging [56]. Tagged CMR can assess regional myocardial mechanics at different time-points during the cardiac cycle [66]. Reduced early diastolic strain rates are present in hypertrophied segments [67]. CMR tagging shows decreased relaxation rate and diminished early diastolic filling velocity as a result of delayed and prolongation of diastolic untwisting [66].

### Assessment and quantification of myocardial fibrosis

Myocardial fibrosis or scaring detected by CMR occurs in up to 33-86% of patients with HCM [30,53,68,69]. LGE-CMR characteristics are not specific for HCM, but the regional location of diffuse LGE within the septum is very suggestive of HCM [16].Table 3 shows incidence of LGE in multiple studies focused on HCM. The weighted mean reported prevalence of LGE, in 1814 LVH positive HCM patients, is 65% (Table 3).

**Table 3:** Incidence of late gadolinium enhancement (LGE) in HCM by CMR

Author	Year	Journal	Number of patients	% of patients with LGE
O'Hanlon et al. [73]	2010	Journal of the American College of Cardiology	217	63%
Bruder et al.[69]	2010	Journal of the American College of Cardiology	220	67.2%
Ho et al. [75]	2010	The New England Journal of Medicine	28	71%
Rubinshtein et al. [111]	2010	Circulation Heart Failure	424	56%
Kown et al. [68]	2009	Journal of the American College of Cardiology	60	63%
Rudolph et al. [76]	2009	Journal of the American College of Cardiology	36	72%
Maron et al. [84]	2008	Circulation Heart Failure	202	55%
Adabag et al. [70]	2008	Journal of the American College of Cardiology	177	40.6%
Kwon et al. [112]	2008	International J. of Cardiovascular Imaging	68	57%
Abdel Aty et al. [93]	2008	Journal of Magnetic Resonance Imaging	27	33%
Paya et al. [113]	2008	Journal of Cardiac Failure	120	69%
Melacini et al. [114]	2008	International Journal of Cardiology	44	80%
Kim et al. [115]	2008	Journal of Magnetic resonance Imaging	25	84%
Debl et al. [116]	2006	Heart	22	73%
Soler et al. [117]	2006	Journal of Computed Assisted Tomography	53	56.6%
Teraoke et al. [82]	2004	Magnetic Resonance Imaging	59	76.3%
Bogaert et al. [83]	2003	American Journal of Roentgenology	11	63.6%
Choudhury et al. [71]	2002	Journal of the American College of Cardiology	21	81%

Summary LGE reports:		Late enhancement	1814	65% [range, 33-84%]

The prognostic significance of the *presence *of LGE in HCM to adverse outcome is high. The presence of LGE in HCM patients has been associated with sudden cardiac death, systolic dysfunction and nonsustained ventricular tachycardia [69-73]. Table 4 lists studies that have evaluated the relationship between presence of LGE and various outcome parameters. Although the *extent *(rather than the *presence*) of fibrosis also was a predictor of major arrhythmic events, the *degree *of fibrosis has not yet been a significant predictor of events in multivariate analysis [73], possible due to small number of study subjects with events. Pathologically, fibrotic tissue is thought to be associated with re-entrant ventricular arrhythmia as well as myocardial dysfunction. The significance of LGE appears high, and larger, multi-center longitudinal trials to assess its prognostic significance seem warranted at this time.

**Table 4:** Relationship between presence of late gadolinium enhancement (LGE) and clinical cardiac events in HCM

Study	Number of patients	% of study population with LGE	Event Rate Positive LGE versus Negative LGE groups	Hazard ratio (HR) or relative risk (RR)	Primary endpoint
O'Hanlon et al. 2010 [73].	217	63	24.5% LGE+ vs. 9.9% LGE- had primary endpoint	HR 3.4 [1.4-8.1]	Cardiovascular death, unplanned cardiovascular admission, sustained ventricular tachycardia or ventricular fibrillation, appropriate implantable cardioverter-defibrillator discharge
Rubinshtein et al. 2010[111].	424	56	3.3% of LGE+ patients had SCD/ICD	n/a	Sudden cardiac death and appropriate implanted cardioverter defibrillator (ICD) discharge
Bruder et al. 2010 [118]	220	67.2	94% LGE+ vs. 66% LGE- had primary endpoint	HR 8.0 [1.0-61.9]	Sudden cardiac death and appropriate implanted cardioverter defibrillator (ICD) discharge.
Adabag et al. 2008 [70]	177	40.6	28% LGE+ vs. 4% LGE- w/NSVT	RR 7.3 [2.6-20.4]	Nonsustained ventricular tachycardia (NSVT) by Holter monitor
Paya et al. 2008 [113]	120	69	38% LGE+ vs. 8% LGE- had NSVT (p < 0.05)	not indicated	Nonsustained ventricular tachycardia (NSVT) by Holter monitor
Suk et al. 2008 [119]	25	64	88% LGE+ vs. 53% LGE- had VT (p < 0.05)	not indicated	Ventricular tachycardia by Holter or resting ECG

HF: Heart failure

ICD: intra-cardiac defibrillator.

NSVT: Non-sustained ventricular tachycardia

OR: Odds ratio.

PVCs: Premature ventricular contractions

VT: Ventricular tachycardia

Multiple factors have been proposed in the etiology of myocardial fibrosis in HCM patients, although the true origin has not yet been determined. Moon et al., [74] found greater collagen deposition correlated directly with LGE of the myocardium. Ho et al., [75] studied the PICP: CITP ratio that reflects the balance between collagen synthesis and degradation. They found a significantly higher PICP: CITP ratio in subjects with overt HCM compared to mutation carriers (without LVH) and the control group. The authors hypothesized that in overt HCM, collagen synthesis exceeds degradation, resulting in frank myocardial fibrosis.

Alternatively, LVH and dynamic LVOT pressure gradients may result in potential pressure necrosis causing small-vessel intramural coronary artery disease (SICAD) [21]. Ischemia may result from microvascular disease; increased end diastolic pressure together with the increased demand of LVH might initiate the processes of myocyte death and replacement fibrosis as a repair process [76].

*Stress perfusion CMR *may be performed in same setting as a LGE CMR study [77,78]. The severity of myocardial perfusion defects correlate with areas of maximal wall thickness and the presence of CMR defined fibrosis in patients with HCM [79]. These studies suggest that microvascular abnormalities precede and predispose to the development of myocardial fibrosis. When present, perfusion abnormalities may represent an early risk marker and a possible therapeutic target [80].

#### Patterns of LGE in HCM

**Figure 2:**
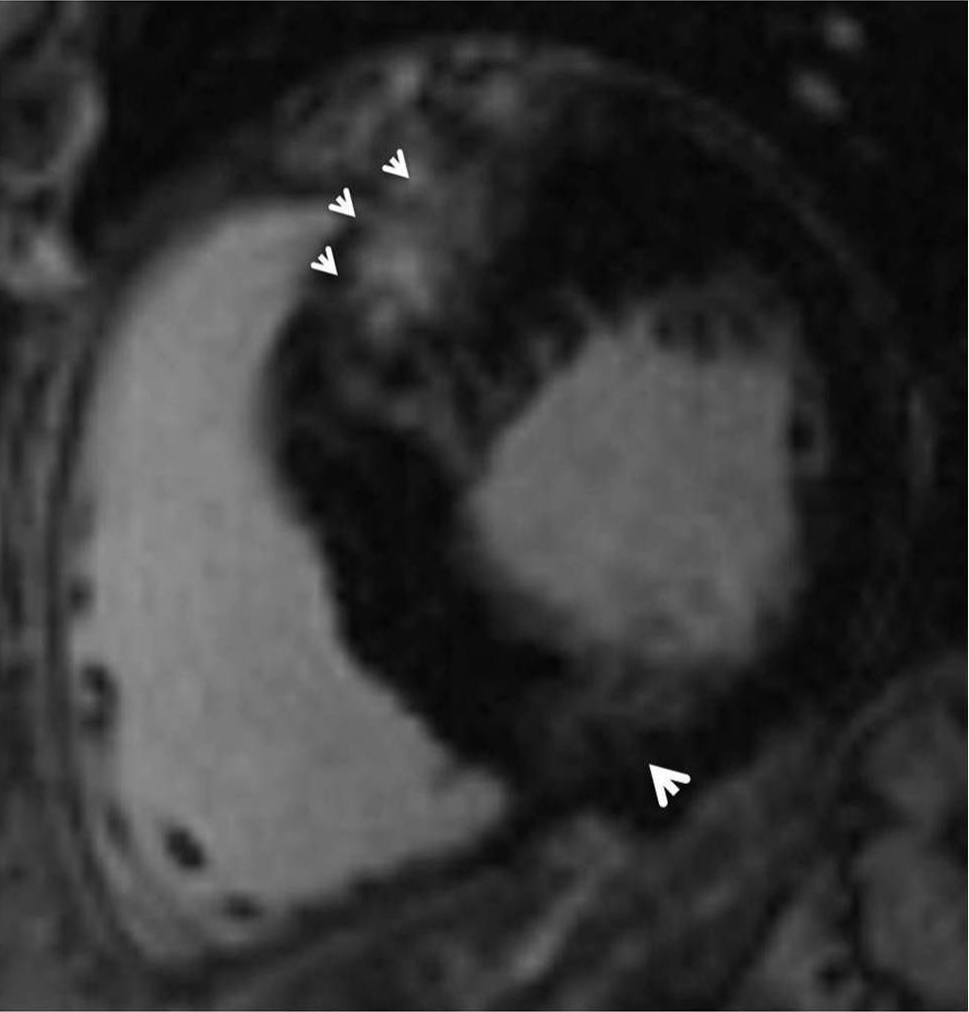
**Late gadolinium enhancement patterns involving the anterior and posterior RV insertion points, as shown; the interventricular septum is involved, particularly the anteroseptal basal segment (three arrow heads)**.

**Figure 3:**
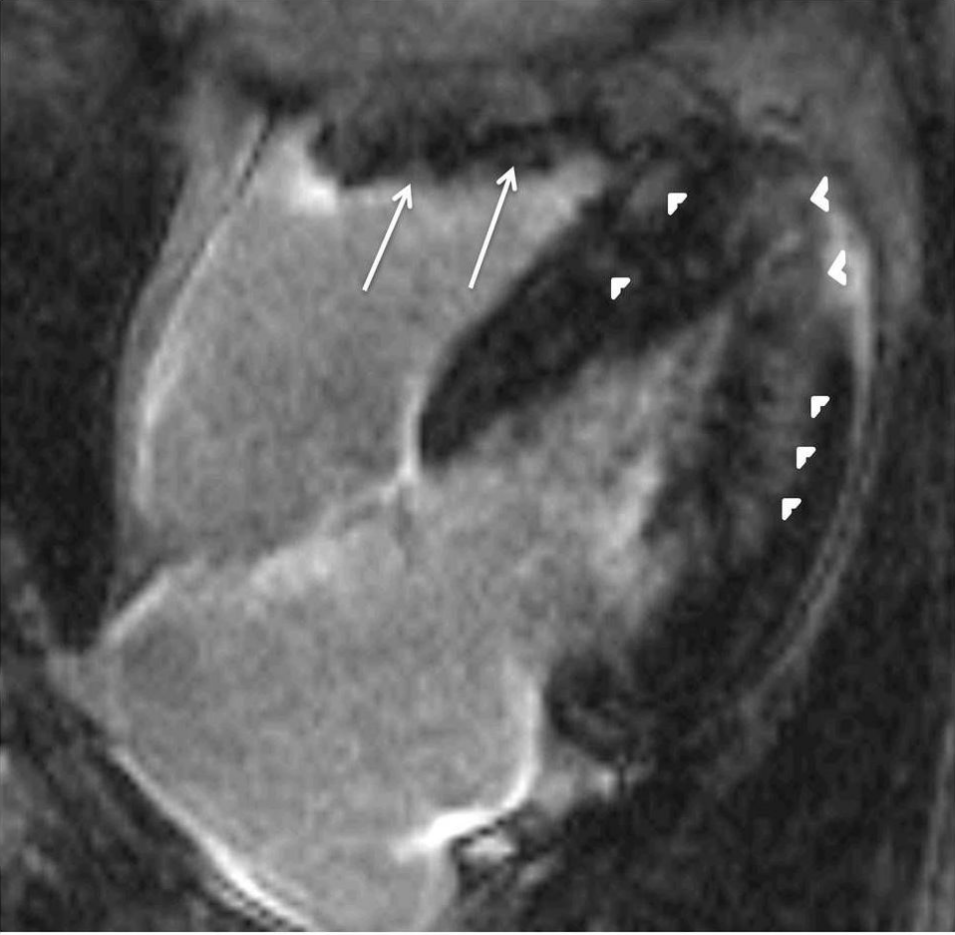
**Patchy mid wall, variable sized foci of hyperenhancement in a non-coronary distribution, involving mainly the hypertrophied parts (arrow heads)**. Right ventricle is also hypertrophied (large arrows).

LGE in HCM characteristically appears as small punctuate, patchy mid-wall hyper-enhancement [29,71]. There is a predilection to occur at the anterior and posterior RV insertion points (Figure 2). These RV insertion points represent plexiform fibrosis containing the crossing-fibers of LV and RV [80-82], although this pattern is not specific for HCM (e.g. patients with right ventricular hypertrophy also may demonstrate LGE at these sites). The interventricular septum is commonly involved by LGE, particularly the anteroseptal mid to basal segments (Figure 2). These segments are also the most commonly thickened segments, especially in patients with asymmetric HCM [81,82]. Other foci of LGE are described as noncoronary in distribution, tending to occur in the hypertrophied regions (Figure 3) [29,71]. An exception to this is areas of burned out HCM where the LV wall is typically thinned and full thickness LGE is present [83].

In a cohort of 202 HCM patients; Maron et al., [84] showed that LGE was most commonly located in both the ventricular septum and LV free wall. Less frequently, LGE was confined to the LV free wall, followed by the septal LGE. The least common sites were the RV insertion into the ventricular septum followed by the LV apex. In patients without LVH, it is unusual to find LGE by CMR. This suggests that LGE occurs after development of LVH [85].

The pattern of LGE may be useful in distinguishing HCM from Anderson-Fabry disease [86,87]. Anderson-Fabry disease is an x-linked disorder of sphingolipid metabolism associated with LVH. In one series, 50% of patients had LGE [86]. The most common location of LGE was in the basal inferolateral wall (12/13 patients) in a non ischemic pattern. The basal inferolateral wall is an infrequent location of isolated LGE for HCM.

#### Functional implications of late gadolinium enhancement

The presence and extent of LGE in relation to impaired ejection fraction (EF) is unclear. Some investigators found no significant relationship [76], but others have shown percent of LV involvement by LGE inversely correlates with LV function [84]. LGE may be associated with increased myocardial stiffness and LV adverse remodeling leading to cavity dilatation and eventually systolic dysfunction [72]. A larger extent of LGE is associated with higher incidence of regional wall motion abnormalities [35,74]. In the presence of LVOT obstruction, there was no significant relationship between extent of LGE and magnitude of LV outflow gradient at rest [76,84].

#### Quantification of diffuse myocardial fibrosis

High-resolution T1 mapping is an emerging technique for detection and quantification of subtle myocardial fibrosis. Abnormal T1 longitudinal relaxation may be useful to quantify the pathologic state of the tissue [88,89]. T1 times are not specific for individual tissues, but each tissue has a normal range that varies by field strength [88,90]. In patients with heart failure, myocardial segments with diffuse fibrosis showed shorter T1 time compared to segments with no LGE [91]. This technique may have potential for evaluation of HCM patients.

Following gadolinium administration in patients with HCM, Amano et al. [92] confirmed that T1 times of hyperenhanced regions of myocardium were lower than remote regions in the same patients. However, the remote regions without visually detected hyperenhancement had altered T1 time compared to a normal control population. This study suggests that T1 mapping may have a role in quantitative characterization of myocardial tissue in HCM. Since the method may detect diffuse fibrosis that is not visually detected, T1 mapping may be applicable to subclinical HCM, discussed further below.

### T2 weighted sequences in HCM

High T2 signal intensity has been reported in HCM patients, frequently corresponding to LGE regions in HCM cases [93]. High T2 signal related to edema may accompany focal ischemia due to SICAD [94,95]. The spatial relation between high T2 signal intensity and LGE could be explained by the pathologic progression of fibrosis: this may start with acute ischemia or inflammation (high T2 signal), and ending with mature chronic fibrous tissue (no obvious T2 abnormalities) [93,96].

### Other CMR applications in HCM

Myocardial disarray can be quantified with *diffusion CMR *[97], either by measuring the myocardial diffusion anisotropy, which is directly correlated with myocardial disarray, or by measuring myofibril orientations [98]. Tseng, et al. [99] found significant reduction of diffusion anisotropy in the hypertrophied septum due to an increase in the longitudinally oriented fibers. Rippplinger et al. evaluated a transgenic rabbit model of human hypertrophic cardiomyopathy. They reported that myocardial fiber anisotropy was similar in the wild type compared to the transgenic animal models [100]. Limitations of diffusion CMR include limited spatial resolution, motion artifact and long imaging times.

**CMR spectroscopy **can be used to measure the ratio of PCr/ATP that reflects the energy status of the myocardial tissue. Decreased PCr/ATP was associated with the extent of hypertrophy and disturbed diastolic function in both symptomatic [101] and asymptomatic patients [102]. Shivu et al. showed PCr/ATP ratio was significantly reduced in HCM patients compared to controls [103]. Perhexiline, a modulator of substrate metabolism, was shown to significantly increase PCr/ATP ratios in HCM patients [104]. Current limitations of CMR spectroscopy including low spatial resolution, long scan time and special coils.

### Role of CMR in early detection of negative phenotypes (pre-clinical HCM)

For the reason that SCD may be the first symptom of HCM [105], there is substantial interest in early identification of HCM mutation carriers who may be at risk for life-threatening events [72,82]. Patients with pre-clinical HCM are negative for LVH, but are genetically positive for the disease. Because CMR accurately presents both anatomic and functional characteristics of HCM, its role in pre-clinical HCM is potentially relevant. To date, most imaging studies of pre-clinical HCM have been based on echocardiography.

Germans et al. [6] evaluated asymptomatic HCM carriers by CMR. They described LV crypts, best visualized at end-diastole, even when LV wall thickness was normal. However, Maron et al.[106] also described a deep LV crypt penetrating 12 mm into a hypertrophied basal posterior portion of ventricular septum of a patient with overt HCM. The significance of LV crypts in HCM remains unclear.

Diastolic function assessed by echocardiography has been reported to be abnormal in pre-clinical HCM [107]. This finding has not been studied by CMR. In addition, tissue characterization by MR spectroscopy or T1 mapping is yet to be explored in preclinical HCM.

## Conclusions

CMR appears to be highly relevant in the clinical as well as research evaluation of patients with overt as well as pre-clinical HCM. Late enhancement after gadolinium administration allows tissue characterization of myocardial fibrosis. The method may potentially identify HCM patients at greatest risk for adverse cardiac events. CMR evaluation of HCM mutation carriers in an early stage of disease has yet to be extensively evaluated, but represents a promising method for exploring the inter-relationship between functional, morphologic and tissue abnormalities in HCM.

## List of abbreviations

AS: atrial systole; BRISK: block regional interpolation scheme of k-space; CITP: C-terminal telopeptide of type I collagen CMR cardiovascular magnetic resonance imaging; CV: cardiovascular; CVA: cardiovascular accident; DD: diastolic dysfunction; LGE: late gadolinium enhancement; LGE CMR: late gadolinium enhancement cardiovascular magnetic resonance; E: early filling; EF: ejection fraction; HCM: hypertrophic cardiomyopathy; HF: heart failure; ICD, implantable cardioverter-defibrillator; LA: left atrium; LV: left ventricle; LVH: left ventricular hypertrophy; LVOT: left ventricular outflow tract; PC: phase contrast; PFR: peak filling rate; PICP: C-terminal propeptide of type I procollagen; RV: right ventricle; SAM: systolic anterior motion; SCD: sudden cardiac death; SERCA: sarcoplasmic reticulum Ca2+ ATPase; SICAD: small-vessel intramural coronary artery disease; VF: ventricular fibrillation; VT: ventricular tachycardia.

## Competing interests

The authors declare that they have no competing interests.

## Authors' contributions

RA carried out the review of literature, manuscript design and drafting, SL has been involved in drafting the manuscript, MN participated in manuscript design and revision, DJ participated in revision of data regarding genetics section, MH carried out the revision of data regarding pathology section, TA has been involved in revising the manuscript critically for important intellectual content, CH has been involved in revising the manuscript critically for important intellectual content, and DB carried out the final revision and approved the manuscript to be submitted.

All authors read and approved the final manuscript.
